# Classifying breast cancer using multi-view graph neural network based on multi-omics data

**DOI:** 10.3389/fgene.2024.1363896

**Published:** 2024-02-20

**Authors:** Yanjiao Ren, Yimeng Gao, Wei Du, Weibo Qiao, Wei Li, Qianqian Yang, Yanchun Liang, Gaoyang Li

**Affiliations:** ^1^ College of Information Technology, Smart Agriculture Research Institute, Jilin Agricultural University, Changchun, Jilin, China; ^2^ College of Computer Science and Technology, Jilin University, Changchun, China; ^3^ School of Computer Science, Zhuhai College of Science and Technology, Zhuhai, China; ^4^ Translational Medical Center for Stem Cell Therapy and Institute for Regenerative Medicine, Shanghai East Hospital, Bioinformatics Department, School of Life Sciences and Technology, Tongji University, Shanghai, China

**Keywords:** multi-view graph neural network, multi-omics data, attention mechanism, feature selection, cancer differentiation, cancer subtypes

## Abstract

**Introduction:** As the evaluation indices, cancer grading and subtyping have diverse clinical, pathological, and molecular characteristics with prognostic and therapeutic implications. Although researchers have begun to study cancer differentiation and subtype prediction, most of relevant methods are based on traditional machine learning and rely on single omics data. It is necessary to explore a deep learning algorithm that integrates multi-omics data to achieve classification prediction of cancer differentiation and subtypes.

**Methods:** This paper proposes a multi-omics data fusion algorithm based on a multi-view graph neural network (MVGNN) for predicting cancer differentiation and subtype classification. The model framework consists of a graph convolutional network (GCN) module for learning features from different omics data and an attention module for integrating multi-omics data. Three different types of omics data are used. For each type of omics data, feature selection is performed using methods such as the chi-square test and minimum redundancy maximum relevance (mRMR). Weighted patient similarity networks are constructed based on the selected omics features, and GCN is trained using omics features and corresponding similarity networks. Finally, an attention module integrates different types of omics features and performs the final cancer classification prediction.

**Results:** To validate the cancer classification predictive performance of the MVGNN model, we conducted experimental comparisons with traditional machine learning models and currently popular methods based on integrating multi-omics data using 5-fold cross-validation. Additionally, we performed comparative experiments on cancer differentiation and its subtypes based on single omics data, two omics data, and three omics data.

**Discussion:** This paper proposed the MVGNN model and it performed well in cancer classification prediction based on multiple omics data.

## 1 Introduction

Cancer is one of the leading causes of death in the world today. According to the global cancer statistics report in 2020, there were nearly 19.3 million new cases of cancer and 10 million cancer-related deaths worldwide (Bray et al., 2018). Due to factors such as globalization and economic growth, the number of new cancer cases is expected to continue to rise. Cancer is a disease characterized by the uncontrolled growth and spreading of specific cells in the body to other parts of the body. These cells can also transfer to distant body parts, forming new tumors through metastasis (Hanahan and Weinberg, 2011). Tumors can be classified into different grades, known as tumor grading, by examining tumor cells under a microscope. Tumor grading compares the degree of cellular and tissue morphological changes between cancer cells and normal cells, indicating the tumor’s differentiation. Generally, based on the abnormality of tumor cells observed under a microscope, tumors are classified into grades 1, 2, or 3 (sometimes also 4), called G1, G2, G3, and G4, respectively (Sobin and Fleming, 1997). These represent well-differentiated, moderately differentiated, poorly differentiated, and undifferentiated tumors. Cancer is also a heterogeneous disease that encompasses various subtypes. The same type of cancer can be divided into subtypes based on different mechanisms of occurrence. Different subtypes of the same cancer reflect distinct molecular carcinogenesis processes and clinical outcomes. With the advent of precision medicine, cancer classification has gradually become one of the fundamental goals of cancer informatics. Heterogeneous cancer populations are grouped into clinically meaningful subtypes based on the similarity of molecular spectra.

Breast cancer is a most common cancer worldwide (Loibl et al., 2021). The number of breast cancer patients is increasing year by year, and the proportion of women under the age of 40 with breast cancer has reached 6.6% (Assi et al., 2013). Breast cancer incidence rates have risen in most of the past four decades; during the most recent data years (2010–2019), the rate increased by 0.5% annually (Giaquinto et al., 2022). Breast cancer, as a highly heterogeneous disease, is composed of different biological subtypes, which possess distinct clinical, pathological, and molecular characteristics, as well as prognostic and therapeutic significance (Reis-Filho and Pusztai, 2011).Therefore, studying breast cancer subtypes is of great significance for precision medicine and prognosis prediction (Waks and Winer, 2019).In the year 2000, Perou et al. first proposed the molecular subtyping of breast cancer. They concluded that breast cancer can be divided into four subtypes: Luminal A subtype, Basal-like subtype, HER2-enriched subtype, and Normal-like subtype (Perou et al., 2000). Sorlie et al. subdivided the luminal subtype into luminal A and B subtypes (Sorlie et al., 2003). Waks et al. categorized breast cancer into three major subtypes based on the presence or absence of molecular markers, including estrogen receptor (ER), progesterone receptor (PR), and human epidermal growth factor receptor 2 (HER2). These subtypes are ER+/PR+/HER2- (luminal A), HER2-positive, and triple-negative breast cancer (TNBC), where all three of these molecular markers are negative (Yersal and Barutca, 2014). The HER2-positive subtype can be further divided into ER+/PR+/HER2+ (luminal B) and ER-/PR-/HER2+. Tao et al. categorized breast cancer into five subtypes based on immunohistochemistry (IHC) markers, including ER, PR, and HER2 (Tao et al., 2019). These subtypes include luminal A, B, HER2-positive, TNBC, and unclassified.

With the advancement of sequencing technologies, various types of omics data in the biosphere, including transcriptomics data [RNA expression data (Wang et al., 2009; Ozsolak and Milos, 2011)], metabolomics (Shulaev, 2006) data, proteomics (Altelaar et al., 2013) data, methylation patterns (Laird, 2010) data, as well as genomics data [DNA sequence data (Metzker, 2010)], have experienced rapid growth and accumulation. Many researchers have developed corresponding tools to handle this large-scale omics data. Another issue gradually gaining attention from researchers is whether there is interaction between complex traits and omics data. Previous studies mainly focused on the relationship between individual omics data and biological processes. Due to the reliance on a single type of omics data in analyzing the causes of complex traits, there have been few research results in this area until now. Through many existing experimental studies, it is known that there is a specific connection between different omics data, and they can complement each other’s missing information. This is crucial for researchers to discover the relationship between complex traits and different omics data (Reif et al., 2004; Sieberts and Schadt, 2007; Hamid et al., 2009; Hawkins et al., 2010; Holzinger and Ritchie, 2012). Integrating different types of omics data and designing reasonable and adequate multi-omics data integration methods to accurately predict cancer differentiation and subtype classification have become hot topics in cancer research.

Deep learning, as an emerging and efficient method in the field of machine learning, is more capable of capturing non-linear complex relationships in complex models. It has been widely used in the research of multi-omics data fusion methods (Cai et al., 2022). Mohammed et al. proposed a LASSO based 1D-CNN method and compared it with SVM, ANN, KNN, and bagging tree methods, the results indicating that the classification performance of the deep stacking method was superior to the traditional machine learning method (Mohammed et al., 2021). Li et al. proposed the MoGCN method by integrating multi-omics data based on a graph Convolutional network (GCN). Autoencoders and similarity network fusion methods are used to reduce and construct a patient similarity network (PSN) respectively to capture complex nonlinear relationships among multi-omics data (Li et al., 2022). Xing et al. Proposed the MLE-GAT method, namely multi-layer embedded graph attention method, uses WGCNA method to format each patient’s omics data into a co-expression network and uses the full gradient map significance mechanism to identify disease-related genes (Xing et al., 2021). Blanco et al. points out the need to maintain a certain balance between biology and computer technology, and to integrate biological knowledge into modeling methods (Linares-Blanco et al., 2021). Leng et al. suggests that the best foundational model for predicting the fusion of multiple omics data is the GNN model (Leng et al., 2022).

This paper considers the relations between feature nodes in the aggregation of GCN model, which are constructed based on multiple sets of omics data to form a similarity network. The correlation between samples can be captured through this similarity network, effectively preserving the biological semantic and geometric structures of the data. While for the GAT model, the relations between nodes are learned through network training. However, especially when the sample size is small, the training effect may not be satisfactory. Therefore, this paper adopts the GCN model instead of the GAT model in the design, and subsequent experiments have also validated this design.

## 2 Materials and methods

### 2.1 Data collection

The breast cancer data used in this study were obtained from The Cancer Genome Atlas (TCGA) database (Weinstein et al., 2013), which contains various cancer types and their corresponding omics data. A total of 606 breast cancer cases were carefully selected, which included gene expression data, DNA methylation data, copy number variation (CNV) data, differentiation annotation, and subtype annotation. The specific statistical information of the mRNA, DNA methylation, and CNV data for the collected breast cancer cases is shown in Table 1. Among the breast cancer cases with differentiation annotation, there were 245 samples labeled as low differentiation (G3), 286 samples labeled as medium differentiation (G2), and 75 samples labeled as high differentiation (G1). The detailed information is presented in Table 2.

**TABLE 1:** Statistics of breast cancer data.

Data type	Number of samples	Number of features
mRNA	606	13195
DNA methylation	606	14285
CNV	606	15186

**TABLE 2:** Statistical information of breast cancer data differentiation.

Breast cancer differentiation	Number of samples
G1	75
G2	286
G3	245

In this article, Tao et al. classified breast cancer into four subtypes using immunohistochemistry (IHC) labeling: luminal A, luminal B, HER2-positive, and triple-negative breast cancer (TNBC). The luminal A subtype is the most common, accounting for 60% of all breast cancer subtypes (Malhotra et al., 2010). The majority of patients with the luminal B subtype are elderly. Approximately 25% of breast cancer patients are HER2-positive, which is associated with a poorer prognosis. Most patients with HER2-positive advanced breast cancer are likely to have lymph node metastasis in the axillary region. The TNBC subtype is characterized by the absence of estrogen receptor (ER), progesterone receptor (PR), and HER2 (Tao et al., 2019). Compared to other subtypes of breast cancer, TNBC tends to rapidly deteriorate and metastasize.

In the breast cancer cases with subtype annotation, there were a total of 398 cases. Out of these, 277 cases were annotated as Luminal A, 40 were annotated as Luminal B, 11 were annotated as HER2(+), and 70 were annotated as TNBC. Table 3 provides detailed information on these cases. The above three omics data and two annotation files are provided in the Supplementary Material.

**TABLE 3:** Classification of cancer subtypes.

Breast cancer subtype	Number of samples	IHC markers
luminal A	277	ER/PR+, Her2−
luminal B	40	ER/PR+, Her2+
HER2(+)	11	ER/PR−, Her2+
TNBC	70	ER/PR−, Her2−

### 2.2 Data preprocessing

**FIGURE 1:**
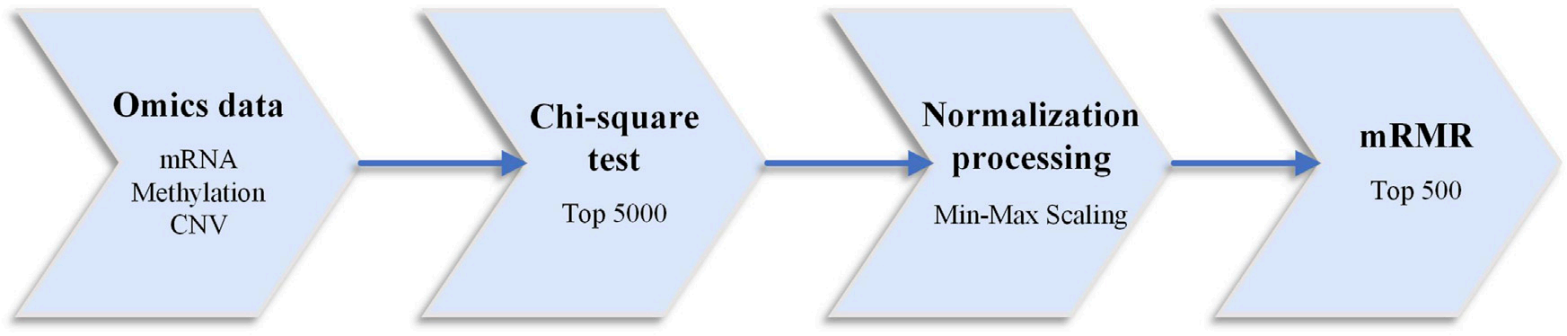
Data preprocessing flowchart.

Generally, deep learning models do not require separate feature selection, as they can achieve this through the neural network’s weights. However, due to the “large p small n” dimensionality catastrophe problem in omics data, training the network weights of omics data using the deep learning model is not adequate. In deep neural networks, fewer features often mean better interpretability and higher training speed. In this study, the collected breast cancer case sample data underwent preprocessing operations using three feature selection algorithms: chi-square test, linear normalization, and minimum redundancy maximum relevance (mRMR) (Yiming, 1997; Peng et al., 2005; Forman, 2008). The specific data preprocessing workflow is shown in Figure 1.

This paper uses the chi-square test to select features for each omics type. The features are sorted based on their number in the hypothesis test using the samples corresponding to each classification task. Then, the top-k features are selected for each omics data. In this study, k is set to 5000. Normalization is performed using linear scaling, transforming the data values to fit within the range of [0,1]. The paper also employs the minimum Redundancy Maximum Relevance (mRMR) feature selection algorithm. The difference between each feature’s maximum relevance value and the minimum redundancy value is used as the feature score. The features are then sorted in descending order based on their scores, and the top 500 features are selected for further filtering. These selected features are favorable for cancer differentiation and subtype prediction.

### 2.3 Graph construction

A graph is a complex data structure consisting of nodes and edges. Many scenes in real life shown in the form of graphs or networks. For example, resources and users in recommendation systems can be considered as nodes in a graph, and the relationships between users and items can be considered as edges. Complex terms like chemical molecules can also be abstracted as graphs (Zhou et al., 2020). Most deep learning algorithms use data such as speech, images, and text with tidy and regular data structures. However, conventional deep learning algorithms are difficult to handle for those irregular and complex network structures. The Graph Convolutional Network (GCN) (Kipf and Welling, 2016) model can process such graph structures.

In this paper, patient similarity networks are constructed by using cosine similarity for three kinds of omics data, namely mRNA, DNA methylation, and CNV data, respectively (Pai and Bader, 2018). The calculation formula for cosine similarity is as Eq. 1:

$$\text{similarity}=\cos\left(\theta \right)=\frac{A\cdot B}{\left\|A\right\|\left\|B\right\|}=\frac{\sum_{i=1}^{n}\;A_{i}\times B_{i}}{\sqrt{\sum_{i=1}^{n}\;{\left(A_{i}\right)}^{2}}\times \sqrt{\sum_{i=1}^{n}\;{\left(B_{i}\right)}^{2}}}$$ (1)

where, A and B are two known attribute vectors, $A_{i}$ and $B_{i}$ respectively represent the components of the vector sum.

Each patient sample is a node in the patient similarity network, and the goal of each GCN in the model is to learn features aggregation from the graph-structured data by leveraging the features of each node and the relationships between nodes. Therefore, the input of the GCN module consists of two parts: the feature matrix and the graph structure description. The feature matrix is represented as $X\in R^{n\times d}$ , where n is the number of nodes and d is the number of input features. The graph structure description is an adjacency matrix $A\in R^{n\times n}$ , constructed by computing the cosine similarity between node pairs. The computation equation is as Eq. 2:

$$A_{ij}=\left\{\begin{array}{cc} s\left(x_{i},x_{j}\right), & \text{ if }i\neq j\text{ and }s\left(x_{i},x_{j}\right)\geq \epsilon \\ 0, & \text{ otherwise } \end{array}\right.$$ (2)

In the equation, $A_{\text{ij}}$ represents the adjacency relationship between node i and node j, $x_{i}$ and $x_{j}$ are the feature vectors of node i and node j, and $s\left(x_{i},x_{j}\right)$ is the cosine similarity between node i and node j. ϵ is a threshold determined by k, where k represents the average number of edges preserved for each node. The computation equation for k is as Eq. 3:

$$k=\sum_{i,j}\;I\left(s\left(x_{i},x_{j}\right)\geq \epsilon \right)/n$$ (3)

where $I\left(∙\right)$ represents an indicator function, and n is the number of nodes. With the similarity network, GCN can be trained using omics features and the corresponding similarity network to learn specific omics data.

### 2.4 Model design

The proposed model in this paper consists mainly of the Graph Convolutional Neural Network (GCN) module and an attention (Velikovi et al., 2017) module. The GCN module is designed for learning the feature aggregation of specific omics data, while the attention module is designed for the fusion of multi-omics features corresponding to different omics data obtained from the output of the GCN module. The attention module can assign different attention weight to each neighbor of a node, thus identifying more important neighbors for better classification of breast cancer differentiation and its subtypes.

**FIGURE 2:**
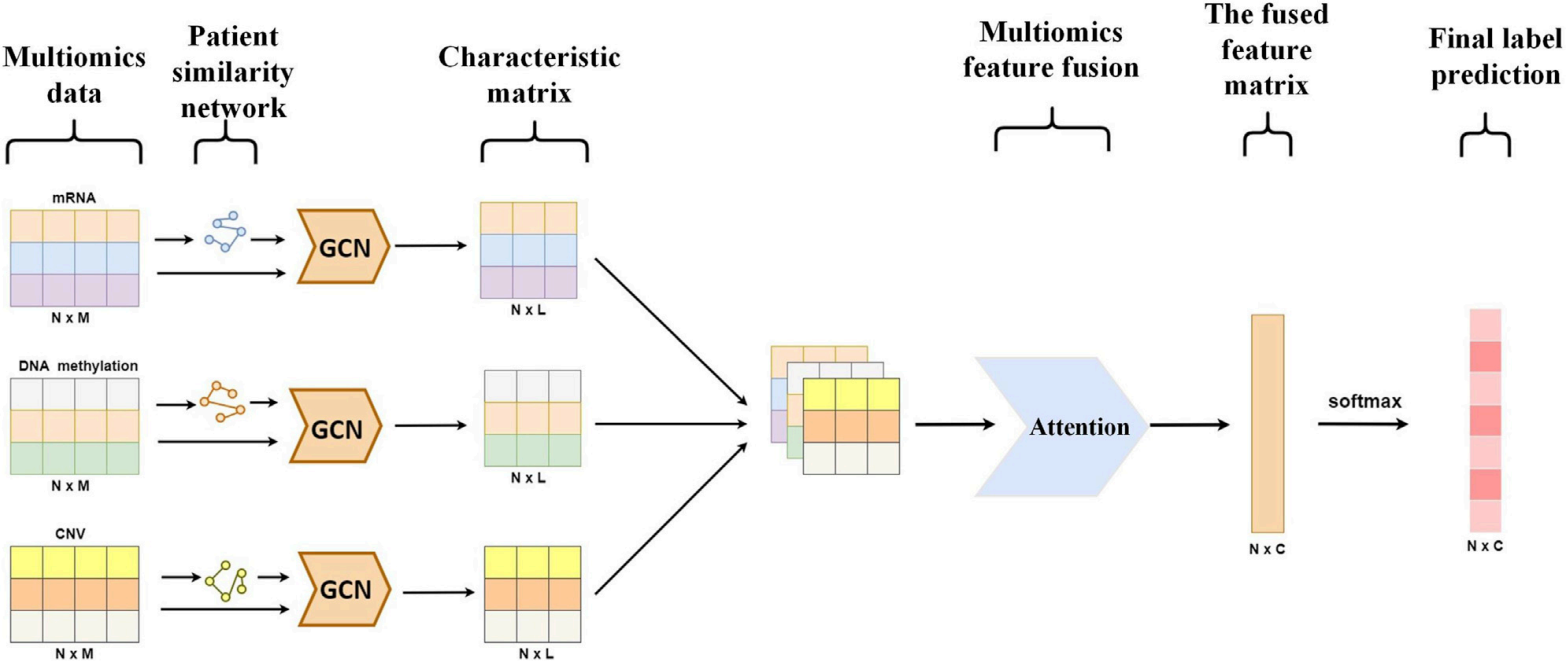
Prediction model of MVGNN.

This paper presents a detailed architecture of the model for predicting the differentiation degree and subtypes of breast cancer, as shown in Figure 2.

In this paper, the GCN is constructed by stacking multiple convolutional layers. Specifically, each layer is defined as Eq. 4:

$$H^{\left(l+1\right)}=f\left(H^{\left(l\right)},A\right)=\sigma \left({\text{AH}}^{\left(l\right)}W^{\left(l\right)}\right)$$ (4)

where l is the number of graph convolutional layers, $H^{\left(l\right)}$ is the input of the l th layer, $W^{\left(l\right)}$ is the weight matrix of the l th layer. $\sigma \left(∙\right)$ represents a non-linear activation function. $H^{\left(l+1\right)}$ is the output of the l th layer. When the number of graph convolutional layers is too large, the resulting node feature vectors will become overly smooth, meaning that the features of each node become very similar. This is mainly because each layer of the GCN integrates information from the node and its neighbors. As the layers deepen, each node incorporates information from more neighbors, including some unrelated nodes. This ultimately leads to similar feature vectors for different types of nodes.

This paper’s model observed that when the number of graph convolutional layers exceeded three, there was no significant improvement in the experimental results. Instead, it increased the computational time and led to overfitting on some datasets. Therefore, the GCN module in this paper’s model consists of three graph convolutional layers.

To effectively train GCN, this paper extends the approach of Kipf et al. (Kipf and Welling, 2016) by further modifying the adjacency matrix A as Eq. 5:

$$\tilde{A}={\hat{D}}^{-\frac{1}{2}}\hat{A}{\hat{D}}^{-\frac{1}{2}}={\hat{D}}^{-\frac{1}{2}}\left(A+I\right){\hat{D}}^{-\frac{1}{2}}$$ (5)

where $\hat{D}$ is the diagonal degree matrix of $\tilde{A}$ , and I is the identity matrix.

The attention model was introduced by Velikovi et al. (2017). The attention model incorporates a self-attention mechanism during the propagation process in the network. Unlike GCN, which treats all neighbors of a node equally, this attention model assigns different attention scores to all neighbors. A higher score for a neighbor indicates a higher importance level for that node. The attention network is implemented by stacking multiple graph attention layers. The input to a single graph attention layer is a set of node feature vectors as Eq. 6:

$$h=\left\{{\vec{h}}_{1},{\vec{h}}_{2},\ldots ,{\vec{h}}_{N}\right\},{\vec{h}}_{i}\in R^{F}$$ (6)

where N represents the number of nodes in the node-set, and F represents the corresponding eigenvector dimension.

The output of each layer is a new set of node feature vectors as Eq. 7:

$$h^{\prime }=\left\{{\vec{h}}_{1}^{\prime },{\vec{h}}_{2}^{\prime },\ldots ,{\vec{h}}_{N}^{\prime }\right\},{\vec{h}}_{i}^{\prime }\in R^{F^{\prime }}$$ (7)

where $F^{\prime }$ represents the new node eigenvector dimension.

In order to obtain sufficient expressive power to transform input features into higher-level features, the graph attention layer first performs self-attention processing according to the set of node feature vectors of input as Eq. 8:

$$e_{ij}=a\left(W{\vec{h}}_{i},W{\vec{h}}_{j}\right)$$ (8)

The shared attention mechanism a is a mapping of $R^{F^{\prime }}\times R^{F^{\prime }}⟶R$ , and $W\in R^{F^{\prime }\times F}$ is a weight matrix that is shared by all ${\vec{h}}_{i}^{\prime }$ . $e_{ij}$ represents the importance of the features of node j to node i .

In this study, the attention module is used to compute the attention coefficients for each omics feature matrix. The attention mechanism is then applied to aggregate different types of omics features, resulting in the final omics feature matrix. The fused feature matrix obtained from the attention module is further processed using SoftMax function for final label prediction.

## 3 Results

### 3.1 Performance metrics

Samples are generally divided into positive and negative classes for binary classification tasks. Therefore, the classifier has four classification results: TP, TN, FP, and FN. TP refers to correctly classifying positive samples as positive. TN refers to correctly classifying negative samples as negative. FP refers to incorrectly classifying negative samples as positive. FN refers to incorrectly classifying positive samples as negative. To evaluate the model’s predictive performance, we mainly used three evaluation metrics: accuracy, F1 score, and area under the receiver operating characteristic curve (AUC-ROC). The specific calculation formulas are as as Eqs 9–14:

$$\text{accuracy}=\frac{TP+TN}{\text{TP}+\text{TN}+\text{FP}+\text{FN}}$$ (9)

$$\text{sensitivity}=\text{recall}=\frac{TP}{TP+FN}$$ (10)

$$\text{specificity}=\frac{TN}{TN+FP}$$ (11)

$$\text{precision}=\frac{TP}{TP+FP}$$ (12)

$$F1\_\text{score}=2\cdot \frac{\;\text{precision}\times \text{recall}\;}{\;\text{precision}+\text{recall}\;}$$ (13)

$$\text{AUC}=\frac{1}{m^{+}m^{-}}\sum_{x^{+}\in D^{+}}\;\sum_{x^{-}\in D^{-}}\;\left(W\left(f\left(x^{+}\right)>f\left(x^{-}\right)\right)+\frac{1}{2}W\left(f\left(x^{+}\right)=f\left(x^{-}\right)\right)\right)$$ (14)

In the paper, “accuracy” refers to the proportion of correctly predicted results among all samples. “F1” is the arithmetic average of precision and recall divided by the geometric mean. F1 has the worst effect when the value is 0 and the best effect when the value is 1. The receiver operating characteristic curve is known as ROC, and the area under the curve (AUC) represents the area under the ROC curve. AUC is calculated through the integral of the ROC curve, and a higher AUC indicates better classification results.

We adopt two evaluation indexes for multi-classification tasks, F1_macro and F1_weighted (Leng et al., 2022). Its calculation formula are as Eqs 15–17:

$$Precision\_macro=\frac{1}{n}\sum_{i=1}^{n}\;{Precision}_{i}$$ (15)

$$Recall\_macro=\frac{1}{n}\sum_{i=1}^{\pi }\;{Recall}_{i}$$ (16)

$$F1\_macro=2\cdot \frac{\;Precision\_macro\times Recall\_macro\;}{\;Precision\_macro+Recall\_macro\;}$$ (17)

F1_macro takes values between 0 and 1 and is unaffected by data imbalance. On the other hand, F1_weighted is the weighted average of F1_score for each category, where the weight is the proportion of each category in the accurate predictions. The difference between F1_weighted and F1_macro is that F1_macro assigns the same weight to each category, while F1_weighted assigns different weights based on the proportion of each category.

The model proposed in this paper and the comparison model are specifically executed on the workstation based on Ubuntu 18.04.5 LTS system and Pytorch v1.7.0. The working environment of the workstation is as follows: CPU is AMD Ryzen 7 3700X 8-Core, 16-Thread,Memory is 64G, GPU is GeForce GTX 1080 Ti (11G).

### 3.2 Implementation details

In deep learning, networks with many parameters are very powerful (Srivastava et al., 2014). However, dealing with the overfitting problem is a key issue. This paper adopts two approaches to address the overfitting issue. The first approach is to add dropout layers to the model. It randomly drops elements in the neural network during training, preventing overfitting caused by excessive training. Each sub-network channel consists of three sequential graph convolution layers and two dropout layers are used in our model and then weighted each channel using the attention mechanism. The second approach is to employ early stopping during the training process of the network model. Specifically, if the loss function of the validation data does not show a significant decrease in the first 100 epochs of training, the model’s training is paused (Prechelt et al., 2012).

This paper computed the cross-entropy between the actual distribution and the predicted distribution of breast cancer differentiation and its subtypes (Tabor and Spurek, 2014). The loss is calculated by minimizing the cross-entropy. The loss function used in this paper’s model is shown in Eq. 18:

$$L=-\sum_{l\in Y_{L}}\;Y^{l}\ln\;\left(C\cdot Z^{l}\right)$$ (18)

where L is the loss function, $Y_{L}$ is the set of node indexes with labels, $Y^{l}$ is the label of the label node, that is, the type of breast cancer differentiation and its subtypes, C is the parameter of the classifier, and $Z^{l}$ is the final node embedding of the label node. This paper optimizes the entire model through end-to-end backpropagation.

### 3.3 The performance of binary classification

#### 3.3.1 Analysis of experimental results of binary classification in differentiation degree

In order to comprehensively evaluate the performance of our MVGNN model compared to traditional machine learning methods and recent supervised multi-omics data integration methods, this paper employs 5-fold cross-validation for different models. The average accuracy, average AUC value, and average F1 value obtained on the test dataset are used as evaluation metrics. These models include Support Vector Machine (SVM), Random Forest (RF), Neural Network (NN), GCN, GAT, and Multi-Omics Graph Convolutional Networks (MOGONET). MOGONET is the latest method for multi-omics data integration published by Wang et al. (2021). The View Correlation Discovery Network (VCDN) are used to explores cross-omics correlations in the feature space, enabling effective multi-omics integration. Three pairs of breast cancer differentiation classifications are considered: well-differentiated vs. moderately-differentiated (G1 vs. G2), well-differentiated vs. poorly-differentiated (G1 vs. G3), and moderately-differentiated vs. poorly-differentiated (G2 vs. G3). The same dataset split is used, and the average accuracy, average AUC value, and average F1 value based on 5-fold cross-validation are used as evaluation metrics. The experimental results of all models in predicting any two types of breast cancer differentiation are shown in Table 4.

**TABLE 4:** The prediction results of classification in any two degrees of differentiation across different models.

Method	ACC	AUC	F1
SVM	0.658	0.645	0.623
RF	0.669	0.649	0.624
NN	0.701	0.674	0.672
NN_NN	0.725	0.708	0.760
NN_VCDN	0.720	0.703	0.752
GCN	0.741	0.704	0.758
GAT	0.749	0.723	0.743
MOGONET	0.744	0.731	0.772
MVGNN	0.778	0.745	0.809

In the experimental process, SVM, RF, NN, GCN, and GAT were trained using preprocessed multi-omics data directly concatenated as input. All methods were trained using the same preprocessed data. According to Table 4, the proposed MVGNN model for integrating multi-omics data achieved the highest accuracy, AUC value, and F1 value compared to traditional machine learning methods, graph convolutional network models, and the latest methods for integrating multi-omics data in classifying any two types of breast cancer differentiation. The values are: accuracy—0.778, AUC—0.745, F1—0.809. It can be concluded that the proposed model in this study outperforms traditional machine learning models and the latest methods for integrating multi-omics data in classifying any two types of breast cancer differentiation.

#### 3.3.2 Analysis of experimental results of binary classification on subtypes

This article adopts a five-fold cross-validation method to train all models, and all methods use the same training set, validation set, and test set. The evaluation metrics are average accuracy (ACC), average area under the curve (AUC), and average F1 score. The classification results of any two subtypes of breast cancer include (1) luminal A vs. luminal B, (2) luminal A vs. HER2(+), (3) luminal A vs. TNBC, (4) luminal B vs. HER2(+), (5) luminal B vs. TNBC, and (6) HER2(+) vs. TNBC. The experimental results of predicting any two subtypes of breast cancer by each model are shown in Table 5.

**TABLE 5:** Prediction results of each model for any two subtypes of breast cancer.

Method	ACC	AUC	F1
SVM	0.7853	0.7725	0.5005
RF	0.8085	0.7917	0.5092
NN	0.8310	0.8103	0.5355
NN_NN	0.8505	0.8433	0.5927
NN_VCDN	0.8417	0.8473	0.6002
GCN	0.8627	0.8457	0.6310
GAT	0.8812	0.8702	0.6405
MOGONET	0.8915	0.9160	0.6632
MVGNN	0.9180	0.9530	0.7155

Based on the data in Table 5, this paper’s model achieved the highest accuracy, AUC value, and F1 score compared to traditional machine learning methods, graph convolutional network models, and the latest integrated multi-omics data methods for any two classification results of breast cancer subtypes. The values are as follows: accuracy - 0.9180, AUC - 0.9530, and F1 score - 0.7155. It can be concluded that this paper’s model outperforms traditional machine learning methods and the latest multi-omics data integration methods in the overall classification results of any two subtypes of breast cancer.

### 3.4 The performance of multi-classification

#### 3.4.1 Analysis of the results of multi-classification experiments on differentiation degree

To better evaluate the performance of the MVGNN model, this paper uses the model to predict the differentiation degree and subtypes of breast cancer based on multi-classification. Specifically, based on the same data set partitioning, this paper uses the average accuracy, average F1_weighted value, and average F1_macro value calculated through 5-fold cross-validation as evaluation metrics. The multi-classification results of breast cancer differentiation degree are G1 vs. G2 vs. G3. The specific experimental results of the MVGNN model and other methods in the multi-classification of breast cancer differentiation degree are shown in Table 6.

**TABLE 6:** Experimental results of multiple classifications of different models in different degrees of differentiation.

Method	ACC	F1_weighted	F1_macro
SVM	0.529	0.5	0.429
RF	0.54	0.532	0.441
NN	0.56	0.547	0.464
NN_NN	0.574	0.549	0.518
NN_VCDN	0.572	0.547	0.506
GCN	0.59	0.575	0.473
GAT	0.608	0.587	0.476
MOGONET	0.6	0.593	0.537
MVGNN	0.621	0.597	0.541

According to Table 6, it can be observed that the MVGNN model proposed in this paper achieves the highest ACC value (0.621), the highest F1_weighted value (0.597), and the highest F1_macro value (0.541) compared to traditional machine learning methods, graph convolutional network models, and the latest integrated multi-omics data methods in the multi-classification results of breast cancer differentiation degree. It can be concluded that the model proposed in this paper outperforms traditional machine learning methods and the latest multi-omics data integration methods in the multi-classification problem of breast cancer differentiation degree.

#### 3.4.2 Analysis of experimental results of multiple classifications on subtypes

In the same way, the experimental details in Section 3.4.1 are utilized in this study. The multi-classification results of breast cancer subtypes are luminal A vs. luminal B vs. HER2(+) vs. TNBC. The specific experimental results of the MVGNN model compared with other methods on multi-classification of breast cancer subtypes are presented in Table 7.

**TABLE 7:** Experimental results of multiple classifications of different models in different subtypes.

Method	ACC	F1_weighted	F1_macro
SVM	0.617	0.627	0.535
RF	0.621	0.635	0.543
NN	0.649	0.633	0.584
NN_NN	0.699	0.679	0.611
NN_VCDN	0.687	0.671	0.609
GCN	0.73	0.721	0.525
GAT	0.733	0.725	0.552
MOGONET	0.712	0.717	0.614
MVGNN	0.735	0.725	0.636

According to Table 7, it can be observed that the MVGNN model proposed in this paper, as compared to traditional machine learning methods, graph convolutional network models, and the latest integrated multi-omics data approaches, achieves the best performance in the multi-classification of breast cancer subtypes. The corresponding performance measures are the accuracy (ACC) value of 0.735, the weighted F1 score (F1_weighted) value of 0.725, and the macro F1 score (F1_macro) value of 0.636. Hence, these results are sufficient to demonstrate the effectiveness of the proposed model in this study.

### 3.5 Ablation experiments

#### 3.5.1 The performance of different network module

• Analysis of experimental results on differentiation classification

To select the module most beneficial for breast cancer differentiation and subtype classification in the model, this study employed a five-fold cross-validation approach to assess the performance of different modules on the same test dataset. For all models, the same training and validation sets were utilized.

**FIGURE 3:**
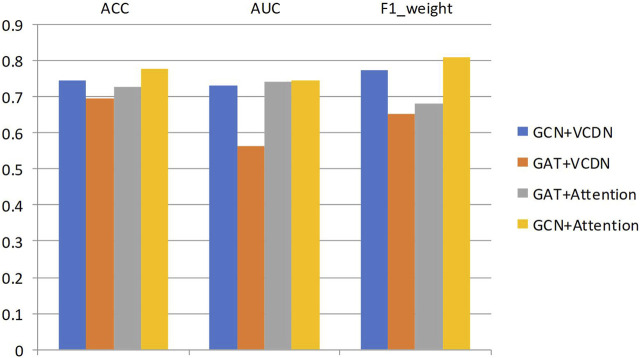
Results of any two classifications of different modules in breast cancer differentiation.

Specifically, this study performed 5-fold cross-validation on the training dataset, with all modules utilizing the same training, validation, and test sets. Mean accuracy, AUC value and mean F1 value were used as measurement metrics. The detailed experimental results of different modules on two types of breast cancer differentiations are presented in Table 8; Figure 3.

**TABLE 8:** Results of any two classifications of different modules in breast cancer differentiation.

Method	GCN + VCDN	GAT + VCDN	GAT + Attention	GCN + Attention
ACC	0.744	0.696	0.726	0.778
AUC	0.731	0.563	0.741	0.745
F1_weight	0.772	0.653	0.682	0.807

By comparing the experimental results of GCN + VCDN and GAT + VCDN, as well as GAT + Attention and GCN + Attention, in predicting any two types of breast cancer differentiations, it can be observed that there exists a specific correlation between biological genomic data. The GAT module did not utilize this correlated information, while the GCN module was able to fully exploit the correlations between biological data, resulting in better differentiation prediction outcomes. Similarly, by comparing the experimental results of GCN + VCDN and GCN + Attention, as well as GAT + VCDN and GAT + Attention, it was found that introducing the attention module improved the performance of predicting breast cancer differentiation. This is because the attention mechanism in the attention module can identify more important neighbors, enabling better classification of breast cancer differentiation. Therefore, this study chose the GCN + Attention model, the MVGNN model, as the final model for predicting breast cancer differentiation.

• Analysis of experimental results on subtype classification

**FIGURE 4:**
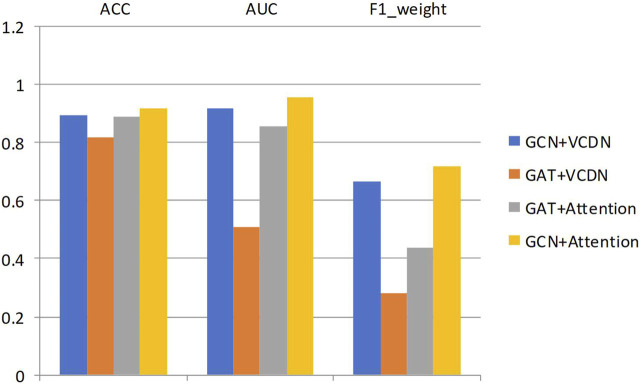
Results of any two classifications of different modules in breast cancer subtypes.

Similarly, the experimental setup for predicting breast cancer differentiation was used. The specific experimental results of different modules on any two breast cancer subtypes are shown in Table 9; Figure 4.

**TABLE 9:** Results of any two classifications of different modules in breast cancer subtypes.

Method	GCN + VCDN	GAT + VCDN	GAT + Attention	GCN + Attention
ACC	0.892	0.818	0.888	0.918
AUC	0.916	0.51	0.854	0.953
F1_weight	0.663	0.283	0.438	0.716

By comparing the experimental results of GCN + VCDN and GAT + VCDN, as well as GAT + Attention and GCN + Attention in predicting two different subtypes of breast cancer, it can be observed that the introduction of the GCN module can improve the accuracy of breast cancer subtype prediction to a certain extent. This is because GCN can effectively utilize the correlation in the biological data. Similarly, by comparing the experimental results of GCN + VCDN and GCN + Attention, as well as GAT + VCDN and GAT + Attention, it can be concluded that the introduction of the attention module increases the precision of predicting breast cancer differentiation. This also indicates that introducing an attention mechanism can improve the model’s performance.

#### 3.5.2 The performance of multi-omics data fusion

• Analysis of experimental results on differentiation classification

**FIGURE 5:**
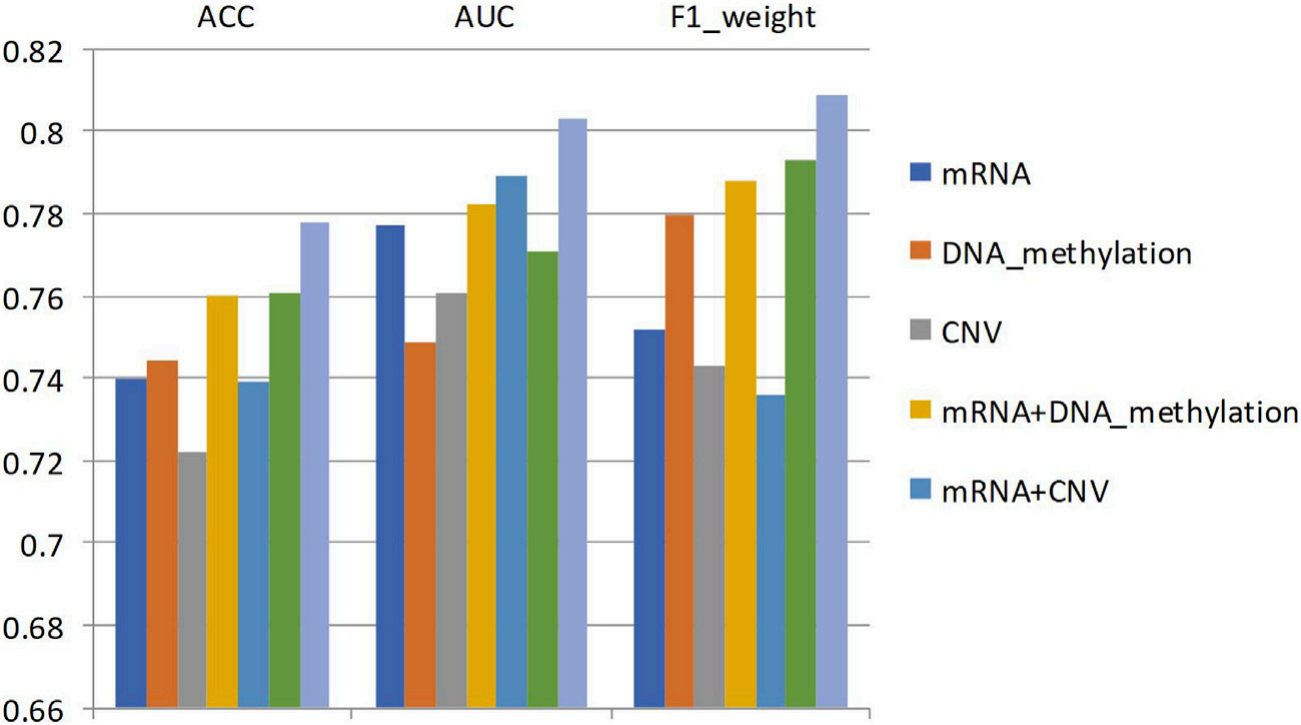
The classification results of any two types of breast cancer differentiation in MVGNN model with different combination of omics data.

Specifically, for different types of omics data combinations, the same data set partitioning was adopted in this study, and the average accuracy, average AUC value, and average F1 value of 5-fold cross-validation were used as metrics. Figure 5 shows the average accuracy, AUC value, and F1 value of the classification results for different degrees of breast cancer differentiation using different types of omics data. DNA_methylation, mRNA, and CNV in the figure represent the single omics data classification experiments using the MvGNN model with mRNA expression, DNA methylation, and CNV data, respectively. mRNA + DNA_methylation, mRNA + CNV, and DNA_methylation + CNV refer to the classification experiments using two types of omics data simultaneously. mRNA + DNA_methylation + CNV refers to the classification experiments simultaneously using all three types of omics data. The specific experimental results are shown in Table 10; Figure 5.

**TABLE 10:** The classification results of any two types of breast cancer differentiation in MVGNN model with different combination of omics data.

Omics data	ACC	AUC	F1_weight
mRNA	0.74	0.777	0.752
DNA_methylation	0.744	0.749	0.78
CNV	0.722	0.761	0.743
mRNA + DNA_methylation	0.76	0.782	0.788
mRNA + CNV	0.739	0.789	0.736
DNA_methylation + CNV	0.761	0.771	0.793
mRNA + DNA_methylation + CNV	0.778	0.803	0.809

From Table 10; Figure 5, it can be observed that compared to using a single type of omics data or combining two types of omics data, the model integrating three types of omics data achieved the highest accuracy AUC, and F1 scores in predicting any two subtypes of breast cancer differentiation. The scores were 0.778, 0.803, and 0.809, respectively. This indicates that the model in this study successfully extracted useful information for classification from different omics data.

• Analysis of experimental results on subtype classification

Similarly, this paper uses the dataset partitioning described in Section 3.5.1 and utilizes the average accuracy, average AUC, and average F1 values from 5-fold cross-validation as performance metrics. Experiments were conducted on the classification of any two subtypes of breast cancer using different types of omics data. The integrated model of three omics data achieved the highest accuracy in classifying any two subtypes of breast cancer, with values of 0.921 (luminal A vs. luminal B), 0.968 (luminal A vs. HER2+), 0.91 (luminal A vs. TNBC), 0.82 (luminal B vs. HER2+), 0.964 (luminal B vs. TNBC), and 0.925 (HER2+ vs. TNBC). This indicates that the model proposed in this paper can extract useful information for classification from different omics data. Furthermore, regarding AUC, the integrated model based on three omics data achieved the highest values in classifying any two subtypes of breast cancer, except for the luminal A vs. HER2+ and luminal A vs. TNBC classifications. The respective AUC values were 0.881 (luminal A vs. luminal B), 0.925 (luminal B vs. HER2+), 0.997 (luminal B vs. TNBC), and 0.979 (HER2+ vs. TNBC). Although the model based on three omics data for the luminal A vs. HER2+ classification was 0.6% lower and for the luminal A vs. TNBC classification was 1.2% lower compared to the models integrating mRNA expression data and CNV data or DNA methylation data, respectively, this still demonstrates the robustness of the proposed model in handling imbalanced samples. Similarly, the model based on three omics data achieved the highest F1 values in classifying two breast cancer subtypes, except for the luminal A vs. luminal B classification. The respective F1 values were 0.36 (luminal A vs. HER2+), 0.799 (luminal A vs. TNBC), 0.58 (luminal B vs. HER2+), 0.973 (luminal B vs. TNBC), and 0.959 (HER2+ vs. TNBC).

## 4 Conclusion and discussion

### 4.1 Conclusion

The grading and subtyping of cancer, as a complex trait with distinct molecular features, has significant prognostic and therapeutic implications. Therefore, cancer grading and subtyping research is essential for precision medicine and prognostic cancer prediction. In recent years, numerous supervised multi-omics data integration methods have emerged domestically and internationally. However, these methods do not consider the interrelationships between different types of omics data, which may lead to a bias towards a specific type of omics data in the final prediction results. It is crucial to explore how to improve the predictive performance of models by utilizing the interrelationships between different types of omics data.

This study proposes a multi-omics data fusion algorithm based on a heterogeneous graph neural network. The algorithm combines graph convolutional networks and graph attention networks to predict the differentiation and subtypes of cancer. The breast cancer data from TCGA is used in this study, which includes gene expression data, DNA methylation data, copy number variation (CNV) data, differentiation level annotations, and subtype annotations for each breast cancer sample.

First, preprocessing operations, including chi-square test, normalization, and minimum Redundancy Maximum Relevance (mRMR), are performed on the three types of omics data for breast cancer. Then, we conduct experiments using the MVGNN model, traditional machine learning algorithms, and popular multi-omics data integration methods separately for binary and multi-class classification of breast cancer differentiation and subtypes using 5-fold cross-validation. According to the experimental results, our model achieves the best performance in both binary classification of breast cancer differentiation and subtypes, and multi-class classification.

Furthermore, to select the modules in the model that are more conducive to predicting breast cancer differentiation and subtypes, we also perform 5-fold cross-validation to test the performance of different modules on the test set. Finally, to further test the classification prediction performance of the model, we compare the differentiation and subtype experiments using only one type of omics data, two types of omics data, and all three types of omics data. Based on the experimental results, the breast cancer classification predictions using the MVGNN model with all three types of omics data perform better than those using two or just one type of omics data.

### 4.2 Discussion

The MVGNN model proposed in this paper has achieved good results predicting breast cancer differentiation and subtypes, but some work will be carried out in future. For example:

The overall classification performance of the proposed MVGNN model is satisfactory. However, from the experimental results in Section 3.5.2, it can be observed that our model needs improvement in differentiating between luminal A and HER2(+) subtypes, as well as between luminal A and TNBC subtypes in breast cancer. This also indicates that our gene expression, DNA methylation, and CNV data are insufficient to distinguish the boundaries between luminal A and HER2(+) subtypes and luminal A and TNBC subtypes. Therefore, there may be differences in these subtypes of breast cancer in other types of omics data. In future work, we aim to integrate additional omics data, such as metabolomics data and mutation data, to enhance our breast cancer subtype classification model.

This paper primarily trains the MVGNN model on the breast cancer dataset from TCGA. In order to further demonstrate the performance of the MVGNN model in cancer classification and diagnosis, future studies can include additional datasets of different cancers, such as lung cancer, liver cancer, gastric cancer, and colon cancer, which have high mortality rates.

## Data Availability

The original contributions presented in the study are included in the article/Supplementary Material, further inquiries can be directed to the corresponding authors.

## References

[B1] Altelaar A. F. Munoz J. Heck A. J. (2013). Next-generation proteomics: towards an integrative view of proteome dynamics. Nat. Rev. Genet. 14 (1), 35–48. 10.1038/nrg3356 23207911

[B2] Assi H. A. Khoury K. E. Dbouk H. Khalil L. E. Mouhieddine T. H. El Saghir N. S. (2013). Epidemiology and prognosis of breast cancer in young women. J. Thorac. Dis. 5, S2–S8. Suppl 1. 10.3978/j.issn.2072-1439.2013.05.24 23819024 PMC3695538

[B3] Bray F. Ferlay J. Soerjomataram I. Siegel R. L. Torre L. A. Jemal A. (2018). Global cancer statistics 2018: GLOBOCAN estimates of incidence and mortality worldwide for 36 cancers in 185 countries. CA a cancer J. Clin. 68 (6), 394–424. 10.3322/caac.21492 30207593

[B4] Cai Z. Poulos R. C. Liu J. Zhong Q. (2022). Machine learning for multi-omics data integration in cancer. iScience 25 (2), 103798. 10.1016/j.isci.2022.103798 35169688 PMC8829812

[B5] Forman G. (2008). “An extensive empirical study of feature selection metrics for text classification,” in Cikm 08: Proceeding of the Acm Conference on Information and Knowledge Mining, Bethesda, Maryland, USA, November 3-7, 1998.

[B6] Giaquinto A. N. Sung H. Miller K. D. Kramer J. L. Newman L. A. Minihan A. (2022). Breast cancer statistics, 2022. CA a cancer J. Clin. 72 (6), 524–541. 10.3322/caac.21754 36190501

[B7] Hamid J. S. Hu P. Roslin N. M. Ling V. Greenwood C. M. Beyene J. (2009). Data integration in genetics and genomics: methods and challenges. Hum. Genomics Proteomics 2009, 869093. 10.4061/2009/869093 20948564 PMC2950414

[B8] Hanahan D. Weinberg R. A. (2011). Hallmarks of cancer: the next generation. Cell 144 (5), 646–674. 10.1016/j.cell.2011.02.013 21376230

[B9] Hawkins R. D. Hon G. C. Ren B. (2010). Next-generation genomics: an integrative approach. Nat. Rev. Genet. 11 (7), 476–486. 10.1038/nrg2795 20531367 PMC3321268

[B10] Holzinger E. R. Ritchie M. D. (2012). Integrating heterogeneous high-throughput data for meta-dimensional pharmacogenomics and disease-related studies. Pharmacogenomics 13 (2), 213–222. 10.2217/pgs.11.145 22256870 PMC3350322

[B11] Kipf T. N. Welling M. (2016). Semi-supervised classification with graph convolutional networks. Available at: https://arxiv.org/abs/1609.02907.

[B12] Laird P. W. (2010). Principles and challenges of genomewide DNA methylation analysis. Nat. Rev. Genet. 11 (3), 191–203. 10.1038/nrg2732 20125086

[B13] Leng D. Zheng L. Wen Y. Zhang Y. Wu L. Wang J. (2022). A benchmark study of deep learning-based multi-omics data fusion methods for cancer. Genome Biol. 23 (1), 171. 10.1186/s13059-022-02739-2 35945544 PMC9361561

[B14] Li X. Ma J. Leng L. Han M. Li M. He F. (2022). MoGCN: a multi-omics integration method based on graph convolutional network for cancer subtype analysis. Front. Genet. 13, 806842. 10.3389/fgene.2022.806842 35186034 PMC8847688

[B15] Linares-Blanco J. Pazos A. Fernandez-Lozano C. (2021). Machine learning analysis of TCGA cancer data. PeerJ Comput. Sci. 7, e584. 10.7717/peerj-cs.584 PMC8293929 34322589

[B16] Loibl S. Poortmans P. Morrow M. Denkert C. Curigliano G. (2021). Breast cancer. Lancet 397 (10286), 1750–1769. 10.1016/S0140-6736(20)32381-3 33812473

[B17] Malhotra G. K. Zhao X. Band H. Band V. (2010). Histological, molecular and functional subtypes of breast cancers. Cancer Biol. Ther. 10 (10), 955–960. 10.4161/cbt.10.10.13879 21057215 PMC3047091

[B18] Metzker M. L. (2010). Sequencing technologies - the next generation. Nat. Rev. Genet. 11 (1), 31–46. 10.1038/nrg2626 19997069

[B19] Mohammed M. Mwambi H. Mboya I. B. Elbashir M. K. Omolo B. (2021). A stacking ensemble deep learning approach to cancer type classification based on TCGA data. Sci. Rep. 11 (1), 15626. 10.1038/s41598-021-95128-x 34341396 PMC8329290

[B20] Ozsolak F. Milos P. M. (2011). RNA sequencing: advances, challenges and opportunities. Nat. Rev. Genet. 12 (2), 87–98. 10.1038/nrg2934 21191423 PMC3031867

[B21] Pai S. Bader G. D. (2018). Patient similarity networks for precision medicine. J. Mol. Biol. 430 (18), 2924–2938. 10.1016/j.jmb.2018.05.037 29860027 PMC6097926

[B22] Peng H. Long F. Ding C. (2005). Feature selection based on mutual information: criteria of max-dependency, max-relevance, and min-redundancy. IEEE Trans. Pattern Analysis Mach. Intell. 27 (8), 1226–1238. 10.1109/TPAMI.2005.159 16119262

[B23] Perou C. M. Sørlie T. Eisen M. B. van de Rijn M. Jeffrey S. S. Rees C. A. (2000). Molecular portraits of human breast tumours. Nature 406 (6797), 747–752. 10.1038/35021093 10963602

[B24] Prechelt L. (2012). “Early stopping — but when?,” in Neural networks: tricks of the trade Editors Montavon G. Orr G. B. Müller K.-R. 2 (Berlin, Heidelberg: Springer Berlin Heidelberg), 53–67.

[B25] Reif D. M. White B. C. Moore J. H. (2004). Integrated analysis of genetic, genomic and proteomic data. Expert Rev. proteomics 1 (1), 67–75. 10.1586/14789450.1.1.67 15966800

[B26] Reis-Filho J. S. Pusztai L. (2011). Gene expression profiling in breast cancer: classification, prognostication, and prediction. Lancet 378 (9805), 1812–1823. 10.1016/S0140-6736(11)61539-0 22098854

[B27] Shulaev V. (2006). Metabolomics technology and bioinformatics. Briefings Bioinforma. 7 (2), 128–139. 10.1093/bib/bbl012 16772266

[B28] Sieberts S. K. Schadt E. E. (2007). Moving toward a system genetics view of disease. Mamm. Genome 18 (6-7), 389–401. 10.1007/s00335-007-9040-6 17653589 PMC1998874

[B29] Sobin L. H. Fleming I. D. (1997). “TNM classification of malignant tumors,” in Union internationale contre le Cancer and the American joint committee on cancer, cancer. 80(9). 5, 1803–1804. 10.1002/(sici)1097-0142(19971101)80:9<1803::aid-cncr16>3.0.co;2-9 9351551

[B30] Sorlie T. Tibshirani R. Parker J. Hastie T. Marron J. S. Nobel A. (2003). Repeated observation of breast tumor subtypes in independent gene expression data sets. Proc. Natl. Acad. Sci. U. S. A. 100 (14), 8418–8423. 10.1073/pnas.0932692100 12829800 PMC166244

[B31] Srivastava N. Hinton G. Krizhevsky A. (2014). Ilya sutskever and ruslan %J journal of machine learning research salakhutdinov. Dropout A Simple Way Prev. Neural Netw. Overfitting 15 (1), 1929–1958. 10.5555/2627435.2670313

[B32] Tabor J. Spurek P. (2014). Cross-entropy clustering, pattern recognition. Available at: https://arxiv.org/abs/1210.5594.

[B33] Tao M. Song T. Du W. Han S. Zuo C. Li Y. (2019). Classifying breast cancer subtypes using multiple kernel learning based on omics data. Genes 10 (3), 200. 10.3390/genes10030200 30866472 PMC6471546

[B34] Velikovi P. Cucurull G. Casanova A. Romero A. Pietro L. Bengio Y. (2017). “Graph attention networks,” in ICLR, Toulon, France, April 24 - 26, 2017.

[B35] Waks A. G. Winer E. P. (2019). Breast cancer treatment: a review. JAMA 321 (3), 288–300. 10.1001/jama.2018.19323 30667505

[B36] Wang T. Shao W. Huang Z. Tang H. Zhang J. Ding Z. (2021). MOGONET integrates multi-omics data using graph convolutional networks allowing patient classification and biomarker identification. Nat. Commun. 12 (1), 3445. 10.1038/s41467-021-23774-w 34103512 PMC8187432

[B37] Wang Z. Gerstein M. Snyder M. (2009). RNA-Seq: a revolutionary tool for transcriptomics. Nat. Rev. Genet. 10 (1), 57–63. 10.1038/nrg2484 19015660 PMC2949280

[B38] Weinstein J. N. Collisson E. A. Mills G. B. Shaw K. R. Ozenberger B. A. Ellrott K. (2013). The cancer Genome Atlas pan-cancer analysis project. Nat. Genet. 45 (10), 1113–1120. 10.1038/ng.2764 24071849 PMC3919969

[B39] Xing X. Yang F. Li H. Zhang J. Zhao Y. Gao M. (2021). “An interpretable multi-level enhanced graph attention network for disease diagnosis with gene expression data,” in 2021 IEEE International Conference on Bioinformatics and Biomedicine (BIBM), Houston, TX, USA, December 9-12, 2021.

[B40] Yersal O. Barutca S. (2014). Biological subtypes of breast cancer: prognostic and therapeutic implications. World J. Clin. Oncol. 5 (3), 412–424. 10.5306/wjco.v5.i3.412 25114856 PMC4127612

[B41] Yiming Y. (1997). “A comparative study on feature selection in text categorization,” in ICML, Nashville, Tennessee, USA, July 8-12, 1997.

[B42] Zhou J. Cui G. Hu S. Zhang Z. Cheng Y. Liu Z. (2020). “Graph neural networks: a review of methods and applications,” in AI open.

